# Renal pelvic involvement in multicentric Castleman disease

**DOI:** 10.1093/rap/rkad108

**Published:** 2023-12-09

**Authors:** Yoshitaka Ueda, Ryohei Nagata, Keigo Setoguchi

**Affiliations:** Department of Rheumatic Diseases, Tokyo Metropolitan Tama Medical Center, Tokyo, Japan; Department of Systemic Immunological Disease, Tokyo Metropolitan Komagome Hospital, Tokyo, Japan; Department of Systemic Immunological Disease, Tokyo Metropolitan Komagome Hospital, Tokyo, Japan

Key message

- CT may demonstrate renal pelvic lesions in multicentric Castleman disease.

Dear Editor, Castleman disease (CD) is a lymphoproliferative disorder first described by Castleman in 1954. Clinically, it is classified into a unicentric or multicentric (MCD) type. Unicentric CD is characterized by lymphadenopathy in a single area and the absence of systemic symptoms. In contrast, MCD is characterized by lymphadenopathy in multiple lymph nodes and a wide spectrum of clinical and laboratory abnormalities. MCD is divided into idiopathic MCD, HHV8-associated MCD and polyneuropathy, organomegaly, endocrinopathy, monoclonal plasma cell disorder, skin changes-associated MCD [1].

MCD can affect various organs, including the kidneys, and previous studies have reported that it can cause renal complications, such as amyloid A amyloidosis, thrombotic microangiopathy or membranoproliferative glomerulonephritis [2]. However, there are few reports of imaging findings of the urological system in MCD. Herein, we report a case of MCD demonstrating rare renal pelvic lesions on enhanced CT.

A 51-year-old male patient received a diagnosis of anaemia at his regular urology clinic. For further investigation of the anaemia, he was referred to another hospital, where laboratory tests found elevated CRP, hypergammaglobulinaemia and elevated serum IgG4. An inguinal lymph node biopsy was performed, and despite the ratio of IgG4/IgG plasma cells being <40%, >10 IgG4-positive plasma cells per high-power field were observed. IgG4-related disease was suspected, and the patient was referred to our hospital for further examination.

He presented with malaise, weight loss, rash and fever. His medical history included hypertension and benign prostatic hyperplasia. His current medical prescriptions included naftopidil, amlodipine, azilsartan, cernilton and fesoterodine. A physical examination revealed blood pressure 133/71 mmHg, pulse rate 117/min and body temperature 37.4°C. Multiple palpable pruritic reddish-brown plaques, each 1–2 cm in diameter, were observed on the extremities and trunk. Lymphadenopathy in the neck and inguinal region was also observed, but the lacrimal and salivary glands were not swollen. The laboratory findings demonstrated white blood cells 8900/μl, haemoglobin 9.5 g/dl, platelets 533 × 10^3^/μl, total protein 12.7 g/dl, albumin 2.3 g/dl, aspartate aminotransferase 12 U/l, alanine aminotransferase 12 U/l, lactate dehydrogenase 116 U/l, blood urea nitrogen 10.3 mg/dl and creatinine 0.74 mg/dl. The patient had polyclonal hypergammaglobulinaemia (IgG 7834 mg/dl; IgG4 1410 mg/dl; IgA 812 mg/dl; IgM 176 mg/dl). Serum and urine tests demonstrated no monoclonal peak on immunoelectrophoresis. CRP, ESR and serum IL-6 were 13.54 mg/dl, 140 mm/h and 44.5 pg/ml (normal: <4.0 pg/ml), respectively. His urinalysis revealed five to nine red blood cells and fewer than one white blood cell per high-power field, and no casts were observed. A serological test for HIV returned negative. Body CT with contrast media revealed multiple centrilobular ground glass opacities in the lungs bilaterally, multiple enlarged lymph nodes in the neck, axilla, supraclavicular area, inguinal region, hilar region and mediastinum, and enhanced soft tissue density surrounding the renal pelvises bilaterally (Fig. 1A and B). We re-evaluated the inguinal lymph node biopsy specimen obtained at the previous hospital, and the histological examination showed sheets of mature plasma cells within the interfollicular zones of the lymph node, which was consistent with MCD (Fig. 1C and D). Given that systemic symptoms, such as fever and elevated inflammatory markers, were considered unlikely to be caused by IgG4-related disease, MCD was diagnosed, and treatment with oral prednisolone 30 mg/day and biweekly i.v. tocilizumab was begun. Follow-up CT demonstrated significant improvement in the pulmonary lesions, lymph node enlargement and renal pelvic lesions. The prednisolone dosage was tapered, and at present the patient is clinically stable at a dosage of 5 mg/day.

**Figure 1.**
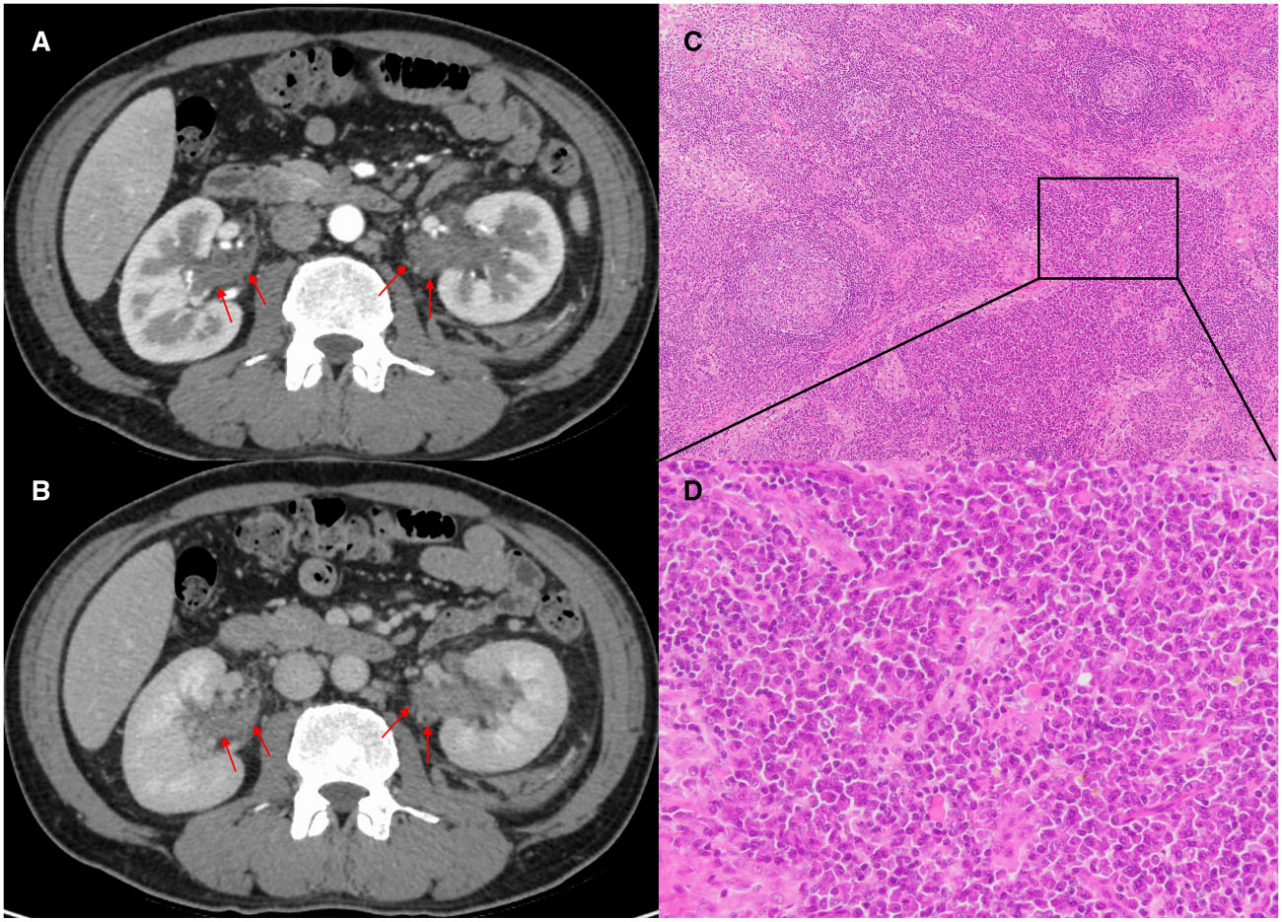
CT images of the renal pelvic lesions and histological image of the inguinal lymph node biopsy specimen. Dynamic contrast-enhanced CT images obtained during the arterial (**A**) and equilibrium (**B**) phases demonstrated renal pelvic lesions (red arrows) with enhancement in the equilibrium phase. (**C**) Lymphatic tissue with preserved follicular structure and enlarged interfollicular zones (Haematoxylin and Eosin staining, ×100). (B) Sheets of mature plasma cells within the interfollicular zones (Haematoxylin and Eosin staining, ×400)

CT findings of MCD usually demonstrate systemic lymphadenopathy, which can be observed at various locations, including the mediastinum, hilar region, neck, axilla and abdomen [3]. On enhanced CT, the lymphadenopathy demonstrates mild to moderate enhancement [4]. Moreover, various pulmonary parenchymal abnormalities, such as centrilobular nodules, thickening of the septa and bronchovascular bundles, may also be observed [5].

Few studies have described imaging findings of urological lesions in MCD. In the present case, soft tissue masses were observed surrounding the bilateral renal pelvises on contrast-enhanced CT, which later resolved with treatment, suggesting that the lesions were associated with the disease. Similar imaging findings have been reported in IgG4-related disease [6, 7], but to the best of our knowledge, there are no reports of such findings in MCD. Given that serum IgG4 levels are often elevated in MCD, it can be challenging to differentiate the latter from IgG4-related disease. Knowledge of urological lesions in IgG4-related disease has grown in recent years, making the diagnosis more challenging in cases with such lesions, and renal pelvic lesions like those observed in the present case can occur in MCD.

## Data Availability

The data pertaining to this case report are available from the corresponding author on reasonable request.
